# Cannabis donation as a harm reduction strategy: a case study

**DOI:** 10.1186/s12954-024-00974-3

**Published:** 2024-03-06

**Authors:** Sarah E. Duhart Clarke, Grant Victor, Pamela Lynch, Leslie W. Suen, Bradley Ray

**Affiliations:** 1 RTI International, 3040 Cornwallis Road, 27709 Research Triangle Park, NC USA; 2 School of Social Work, Rutgers University, 120 Albany Street, New Brunswick, NJ 08901 USA; 3 Division of General Internal Medicine at San Francisco General Hospital, Department of Medicine, University of California San Francisco, 2540 23rd Street, San Francisco, CA USA

**Keywords:** Harm reduction, Cannabis, Overdose prevention, Public health

## Abstract

**Background:**

The United States (US) continues to experience unprecedented rates of overdose mortality and there is increased need to identify effective harm reduction practices. Research from Canada describes cannabis donation through harm reduction agencies as an adjunctive strategy to mitigate the negative consequences of more harmful drugs. This case study describes the operational logistics, feasibility, and potential benefits of a cannabis donation program that was operated through a harm reduction program in rural Michigan.

**Case presentation:**

We applied a community driven research approach to gather information from harm reduction program staff about the implementation and evolution of cannabis donation efforts in Michigan. We also examined 20-months (September 2021 through May 2023) of administrative data from a cannabis company to compare the sale and donation of cannabis products. Ten cannabis-experienced harm reduction clients received cannabis donations, with clinical staff determining client interest and appropriateness, and providing weekly pick-up or delivery. To expand product availability and sustainability, we examined administrative data from a commercialcannabis company that volunteered to provide donations. This administrative data suggests that while flower products constitute most of the adult and medical sales, edible, oil, and topical products predominated donations. Further, cost analysis suggests that donations represent only 1% of total gross sales and account for much less than the expected yearly donation amount.

**Conclusions:**

Research suggests there is potential to reduce alcohol and drug use related harms of more dangerous substances through substitution with cannabis. This case study is the first to document cannabis donation as a harm reduction practice in the US and suggests potential for sustainability dependent on state laws. Findings from this case study provide a starting point for inquiry into cannabis donation as a harm reduction strategy in the US; future research is needed to fully understand the individual-level outcomes, public health impacts, necessary legal regulations, and best practices for cannabis donation programs through harm reduction organizations.

## Background

The overdose epidemic is a persistent and worsening public health problem in the United States (US). It is now the leading factor driving decreased national life expectancy rates [1]. Across multiple waves—from prescription opioids, to heroin, to illicitly manufactured fentanyl (a synthetic opioid 50 to 100 times more powerful than heroin)—overdose rates have continued to increase with recent trends showing mortality increases highest among Black people, Indigenous people, and adolescents [1–3]. As overdose deaths continue to surpass historic rates, there is an increased need to understand the implementation of novel interventions in the US, that aim to reduce harms associated with hazardous substance use. Cannabis as a safe supply harm reduction tool is another example of a novel intervention to address drug-related morbidity and mortality [4].

While there are risks inherent in any drug use, research has long suggested that cannabis has the potential to reduce illicit drug use related harms through substitution [5–10]. Intentional use of cannabis is associated with decreased use of other substances [11–18], from alcohol [19–22] to methamphetamine [23] to fentanyl [24], and in jurisdictions where cannabis is regulated, there is evidence of reductions in overdose deaths [25, 26]. Additionally, medical use of cannabis can augment the treatment of chronic pain [27] and opioid use disorder treatment [28]. For example, a recent study conducted in Canada showed that cannabis use among people who use opioids was associated with reductions in opioid use and was able to alleviate opioid withdrawals, particularly among people living with moderate to severe pain [29]. While the role of cannabis in affecting or treating substance use related disorder requires much further investigation [30, 31], it is reasonable to suggest, particularly in a regulated cannabis market [32], that consuming cannabis is safer than the increasingly toxic illicit drug supply [33].

There have been calls for the implementation of cannabis-based interventions in harm reduction settings [34, 35]. Studies from Canada describe cannabis donation through harm reduction agencies as an adjunctive strategy to mitigate the negative consequences of more harmful drugs [4, 29, 36]. However, the practice of cannabis donation as harm reduction has yet to be studied or described in the US. The goal of the current case study is to document operational logistics, feasibility, and potential benefits of an unsanctioned cannabis donation program implemented through a harm reduction organization in rural Michigan. Specifically, we applied a community driven research (CDR) approach to gather information from program staff about program processes, how current policy affects programming, program participants, and reported outcomes; CDR emphasizes a full collaborative relationship between researchers, people with lived/living experience, and grassroots organizations, in which community partners are active members of the research team [37]. In this paper, we discuss cannabis donation as a harm reduction intervention organically happening among practitioners and a population of people dependent on alcohol, methamphetamine, and/or opioids. We discuss cannabis donation practices within the context of its legal landscape and aim to drive more rigorous inquiry into this potentially lifesaving practice.

## Case presentation

### Study setting

The cannabis donation and distribution program described in this case study was implemented through a harm reduction program in Michigan where cannabis use has been legal for medical use since 2009 and adult “recreational” purposes since 2018 (MLA 333.26424, MLA 333.27955). Caregivers (i.e., growers who register up to five medical patients with the state who are compensated for costs associated with production) have been producing much of the cannabis in Michigan since 2009; though, since 2018, adults over the age of 21 can also grow up to 12 plants for personal use just as large commercial production and sale of cannabis was implemented across the state. There were an estimated 30,000 caregiver in 2021 [38] and practices are regulated by the state and require that persons not have a record of committing a felony and can deliver up to 2.5 ounces for up to five patients. It is common knowledge that caregivers produce more than necessary and under state laws caregivers can gift, donate, or transfer excess product to adults as a gift without sale while individual adults can also transfer up to 2.5 ounces of cannabis (equivalents to one ounce of usable cannabis are 16 ounces of infused product in a solid form, 7 g product in a gaseous form, 36 fluid ounces in liquid form) to another adult as a gift, but the transfer cannot be promoted to the public. This is not a clandestine practice; in fact, the state’s cannabis regulation agency has promoted and supported donation to veterans [39]. It was beyond the scope of the current study to examine other state laws around donation practices.

The harm reduction program passing through cannabis donations in this case study is a non-profit directed by a licensed social worker and certified drug and alcohol counselor. The organization reaches approximately 9,000 clients per year through mobile outreach and services offered at their brick-and-mortar location. During the time reported on in this study, available services included peer recovery support, blood borne pathogen screening, referral, linkage to care, overdose education and naloxone distribution, out-patient substance use disorder therapy, and clinical substance use disorder treatment (including medication for opioid use disorder – MOUD). The information in this case study was gathered by authors during monthly meetings between agency staff, researchers and donors that also included local community members who advised on experiences with prior cannabis donation efforts in the state. Additionally, we examined 20-months (September 2021 through May 2023) of administrative data from a cannabis company to compare the sale and donation of cannabis products.

### Initial program development and donation practices with grassroots donors

While donations of cannabis are legal, promoting the practice is prohibited. Thus, there were no advertisements of the program or published inclusion criteria. Clients did not opt in but were discreetly and confidentially identified by clinical staff, based on clients’ diagnoses and prior or current use of cannabis to reduce mental, emotional (e.g., anxiety, post-traumatic stress disorder) or physical (e.g., back pain, arthritis) problems. Potential clients were then screened for current criminal legal system involvement, as ongoing drug testing by the criminal justice system precluded some interested clients’ ability to participate.

The grassroots cannabis donors (i.e., caregivers) were identified through a volunteer from the research team, who shared information about similar programs in Canada. Three donors were initially identified, all with personal experience using cannabis to mitigate the risk of more harmful substances, who were able to provide edible products and concentration derived from cannabis they produced. As harm reduction clinical staff determined client interest in donated cannabis, they also determined preference among available products and developed an individual plan per client for weekly in-person pick-up or delivery. The cannabis products were kept off-site from the harm reduction agency: volunteers traveled to grassroots donors to receive the products, then traveled to meet agency staff to provide the donation to ensure the transfer was among verified individuals and within the allowed state regulations. The participants then picked up the gifted donations or requested their delivery on a weekly basis. Most participants picked up their gifted donations from the harm reduction agency, while two (sometimes three) participants with limited transportation typically requested delivery.

Donations from caregivers started in August 2020 and occurred throughout the COVID-19 pandemic, without barriers to agency services as harm reduction services were immediately deemed essential by the Michigan Department of Health and Human Services (MDHHS) at the start of the COVID lockdown period (March 2020.) Over a 12-month period (August 2020 through July 2021) these caregivers estimated that they had provided over a thousand edible cannabis products, including nearly 50 containers of tincture and 20 units of flower and concentrate which are smokeable products.

### Description of initial program participants

The harm reduction agency selected ten initial clients to receive cannabis donations, all of whom had a history of using cannabis as an adjunct to other substances (opioids, methamphetamine, and alcohol). The initial group of ten program participants included six women and four men. Participants ranged in age from 24 to 60 years old: three participants were in their 20s, three were in their 30s, two people were in their 40s, and two were in their 50s.

All participants were dependent on alcohol, methamphetamine, or opioids, with many reporting co-occurring dependencies. Nine of the ten had some history of opioid dependency, although one of those nine was in full remission from opioid dependence at the time of the study. Only one of the participants, in their twenties, had a history of alcohol dependence, and long-term cannabis use. Three total participants identified alcohol dependency as the biggest challenge to sobriety at the time of the study. Of the participants with current opioid dependency, five participants reported co-occurring dependencies: four participants had opioid and methamphetamine dependencies; and one participant had alcohol, methamphetamine, and opioid dependencies. It is also noteworthy to mention that the person reporting methamphetamine, alcohol, and opioid dependencies lives in an environment of severe domestic violence. One of the participants in their 20s was pregnant, homeless, and injecting opioids and methamphetamine.

### Evolution of cannabis donation through commercial operations

To assure ongoing donations and expand the variety of donated products, a commercial cannabis producer and distributor was identified by the research team through the existing network of grassroots donors, and offered graciously to provide a consistent, accessible supply and variety of products. This locally owned small business, with a 40,000 square foot indoor cultivation facility and three provisioning stores, sells both their own product line, along with other brands grown or produced in-state, and includes concentrate cartridges, edibles, extracts, flower, tinctures, and topicals. While state law regarding donation is not specific to commercial facilities, they donate by selling products for a penny [39] and a facility agreed to ongoing donation to the harm reduction agency for up to $12,000 in wholesale cost of cannabis products yearly and, unlike donations from caregivers, these products were collected directly from the adult use business location by harm reduction agency staff. The donations were maintained within the quantities allowed by state regulations from the business to agency staff who then made them available to the selected program clients.

Harm reduction agency staff again determined client interest in cannabis products, which now included smokable items and pain topicals from the business and maintained weekly provision. When cannabis products were obtained by the organization, the commercial cannabis producer would track them as part of the sales process. Table 1 compares the adult recreation and medical sales from this cannabis retailer over a 20-month period (September 2021 through May 2023) to donations during this same period. Edibles were the most utilized product (41.6%) among recreational sales while flower was the most popular product for medical sales (33.1%) during the 20-month period. Among donation participants, edibles were the most utilized product (67.9%). Donation participants often used a variety of products, as they were available. Harm reduction staff reported that participants’ high use of edibles was largely due to their consistent availability. That is, many program participants indicated they would prefer flower over edible products if given the option; however, harm reduction agency staff were legally limited in the amount of flower they were able to pick up in each visit to the business, and thus often did not have enough flower to distribute adequate amounts to program participants. It should also be noted that caregivers originally donating to the project pre-dispensary involvement had not donated flower, thus flower had not been an option for the clients until the dispensary got involved. Caregivers largely did not donate flower because many of their patients preferred flower and thus would take most this product; caregivers would then typically use any flower that was not taken by patients to make edibles. In terms of sustainability, the gross sales and cost of the products suggests that donations to these 10 clients represent approximately 1% of total gross sales ($1,400,506 sales and $22,908 donation), costing the business only $8,507 in products over the 20-month period—amounting to 70.1% of what they agreed to donate annually ($12,000). The $8,507 in product costs over the 20-month period amounts to approximately $0.10 in costs-per-client.

**Table 1:** Cannabis units sold compared to those donated from commercial cannabis business by product type (September 2021 through May 2023)

	Adult Sales					Medical Sales					Donations			
*Product Type*	N	%	Sales	Cost		N	%	Sales	Cost		N	%	Sales	Cost
Concentrate	7,298	18.7	$222,377	$99,012		432	19.3	$14,370	$9,246		92	14.5	$2,905	$1,310
Edible	16,232	41.6	$183,949	$83,528		716	31.9	$12,706	$5,486		427	67.2	$5,717	$2,537
Extract	4,748	12.2	$146,722	$64,724		343	15.2	$11,980	$6,674		55	8.7	$1,835	$872
Flower	10,568	27.1	$838,742	$273,943		742	33.1	$88,039	$39,326		51	8.1	$12,054	$3,737
Tincture	156	0.3	$5,065	$2,325		10	0.1	$350	$170		3	0.4	$102	$51
Topical	93	0.1	$3,651	$1,825		32	0.4	$1,464	$684		7	1.1	$289	$144

Notes: N is units of product types sold or donated. Concentrates are cartridges and along with extract and flower are smokable products while edible and tincture are consumable. Sales are gross and costs are based on the wholesale purchase price to commercial cannabis business

### Reported benefits from program participants

When harm reduction agency staff inquired with clients about specific uses of donated cannabis, clients reported decreased amount and/or frequency of using more harmful substances, reduced anxiety, improved ability to manage withdrawal, and an increased ability to sleep. Some individuals reported abstaining from illicit substances and alcohol entirely with cannabis use, while others reported improvements in quality of life and a reduction of cravings for other substances. A couple of anecdotal stories about the impacts of the cannabis donation program for participants are described below.

One of the 50 + year old participants had spinal fusion neck surgery (with the installation of two steel rods, three connectors, and six bolts) five months into the study. Before the surgery, this person had not used opioids for two years (as evidenced by criminal legal mandated urine drug screens) but reported frequent struggles in denying himself alcohol. With their use of the products donated by this program, this individual reported complete abstinence from alcohol while recovering from their surgery and since. They expressed gratitude for topical pain relief with cannabis pain cream, cannabis vape cartridges, and flower for smoking.

One participant in her 20s was pregnant, homeless, and a methamphetamine and opioid dependent injector at the beginning of the study. She reported that with the use of products donated in this program, she used methamphetamine and opioids less frequently, and actively worked with harm reduction agency staff to get on MOUD while pregnant. 

## Discussion

We provide the first documented description of a cannabis donation and distribution program within existing structures of a harm reduction service provider in Michigan, USA. Previously, only Canada had documented this practice in the academic literature [4, 36], with the most recently published study showing that access to cannabis products may be a promising strategy for combatting harms associated with an increasingly toxic illicit opioid supply [29]. While cannabis donation efforts in the US remain unsanctioned, they leveraged state-level cannabis policy so that persons with high risk of overdose could be provided a safe supply of cannabis as an auxiliary harm reduction tool to reduce negative consequences of more harmful substances [40].

This case study shows evidence of the feasibility of a cannabis donation and distribution program. We found that both caregivers and commercial cannabis distributors have the capacity to function as donors and, importantly, there is agreement in trained and licensed clinicians to support the use of cannabis products by those in need. Caregivers operate in a largely unregulated space in Michigan and, based on the findings here, may donate cannabis when they have excess product, especially when they have personal experience using cannabis to mitigate the dangers of other substances. We found that for commercial cannabis distributors who donate, the revenue loss is minimal. For cannabis retailers who are hesitant to donate cannabis products because of concern over revenue loss, this study provides initial evidence for the low impact of donations on total revenue. Additionally, commercial cannabis data revealed that donations represent an incredibly small portion of the products being sold through these facilities, but also that harm reduction agency clients can benefit from products (i.e., edible and/or topical items) that are low in cost, but still out of reach financially for many. Beyond revenue loss, concerns of liability and legal precariousness, along with stigma against people who use illicit drugs (even among users of cannabis) [41–43], may be the key impediment to the expansion of donation efforts. In many states cannabis retailers are required to commit to activities that provide service to the community [44], which may be a facilitator for expanding cannabis donations through harm reduction service providers.

Harm reduction recognizes that when someone cannot or will not stop using dangerous substances, their safety and survival still take precedence. Over the past decade some harm reduction practices have become less stigmatized by federal entities in the US, as demonstrated by ending the ban on funding for syringe service programs and more recently by providing funding for harm reduction services and research as part of a national overdose prevention strategy [45, 46]. Michigan state policy further demonstrates support for harm reduction practices, including naloxone distribution, access to medications for opioid use disorder (MOUD), criminal legal system diversion, and etc. [47]. As cannabis policy evolves, harm reduction agencies need to be included in legal decision-making to mitigate access factors that exclude their clients from the legal market. For example, while the emergence of regulated cannabis markets has provided demonstratable benefits [26], affordability is noted as the main barrier toward use of regulated cannabis products by individuals with substance use disorders [4, 48]. Moreover, despite feasibility of donations demonstrated in this paper and feedback from harm reduction agency staff that the donation program was well-received and helped produce positive outcomes, the time required to facilitate cannabis donation under current regulations can be burdensome, particularly among harm reduction organizations already relying on a skeletal labor force, due to insufficient funding – resulting from stigma toward harm reduction programming and approaches. Following state regulations for donations from caregivers required extensive volunteer time to acquire and transfer cannabis products, while those products obtained from the retail store required harm reduction agency staff time and mileage to travel to the store, maintain products offsite from the agency, and then assure that products were picked up by or delivered to clients. Programmatic and policy reformulations are necessary to address this burden, such as funding harm reduction agency staff time and mileage, increasing the allowable donated amount, creating direct donation linkages between caregivers or commercial cannabis distributors and harm reduction agency staff, or allowing for mail order systems for cannabis donations.

While this is the first study to document the donation of cannabis as a harm reduction practice in the US, it is exploratory and not designed or intended to assess the outcomes associated with this practice. Instead, we focus on describing how this process has been organically occurring in a state where there is the provision of cannabis and statutes that allow for donation. However, this study is not without limitations. For example, while researchers and practitioners partnered to document and understand the donation process, researchers did not interact directly with clients receiving donations. Therefore, detailed individual-level data and information on clients came from harm reduction agency staff. Additionally, it was beyond the scope of the current study to conduct an examination and legal analysis of cannabis donation regulations in other states. It is important to note that the lessons learned in this study may not be generalizable to other harm reduction organizations or states where cannabis is regulated; much more research is needed to examine client perceptions and use patterns, with a focus on understanding whether donating cannabis through harm reduction agencies is creating pathways towards a safe supply and reducing harms from more dangerous substances.

## Conclusions

Despite billions spent at federal, state, and local levels, the US continues to face a drug overdose public health crisis. As illustrated in this case study, cannabis donation through harm reduction is happening in the US. While the policies surrounding the regulation and distribution of cannabis can still present barriers towards this practice, harm reduction staff working in the field see the potential benefits of cannabis, which include reduced premature death [17, 49], improved quality of life [50, 51], pain moderation [29, 52–54], increased recovery outcomes [10, 15, 55, 56], and improved safety for clients and community [57, 58]. Future research should focus on assessing whether this harm reduction practice is achieving any of these outcomes. Until then, given the ongoing overdose mortality stemming from illicitly produced fentanyl and other synthetic contaminants saturating the unregulated drug market, and the potential benefits of cannabis in reducing this unregulated substance use, harm reduction practitioners will continue to support client self-determination, and mutual aid in all forms, including available safe psychoactive substances, for persons who use drugs.

## Data Availability

Not applicable.
